# Biological Therapies in Inflammatory Myopathies

**DOI:** 10.5041/RMMJ.10495

**Published:** 2023-04-30

**Authors:** Abd El Haleem Natour, Shaye Kivity

**Affiliations:** 1 Rheumatology Unit, Meir Medical Center, Kfar Saba, Israel; 2 Department of Internal Medicine A, Meir Medical Center, Kfar Saba, Israel

**Keywords:** Abatacept, bDMARDs, idiopathic inflammatory myopathies, IVIg, JAKI, rituximab

## Abstract

Idiopathic inflammatory myopathies (IIM) are a rare group of disorders that feature progressive immune-mediated skeletal muscle destruction along with skin, lung, and joint involvement. Management of IIMs necessitates glucocorticoid therapy followed by conventional steroid-sparing agents to control disease activity. In the settings of refractory myositis or life-threatening manifestations, e.g. lung involvement or oropharyngeal dysphagia, second-line therapies are needed to minimize disease burden, avoid end-organ damage and steroid toxicity, and decrease mortality. These therapies may include biological disease-modifying antirheumatic drugs (bDMARDs), and to a lesser extent, targeted synthetic disease-modifying antirheumatic drugs (TSD). This article reviews the current use of bDMARDs, e.g. intravenous immunoglobulin and rituximab, and a TSD—Janus kinase inhibitors (JAKI)—along with their indications, efficacy, and safety in managing IIM.

## INTRODUCTION

The idiopathic inflammatory myopathies (IIM) represent a rare group of disorders marked by progressive immune-mediated skeletal muscle destruction together with skin, lung, and joint involvement. Organ involvement and disease severity tend to differ in IIM. While in some cases the disease course can be monophasic and short-lived, IIM generally involves persistent disease activity that waxes and wanes. Studies have shown that specific disease phenotypes often have distinctive serologic laboratory findings that may correlate with the disease course and prognosis of patients with IIM. Treatment is based on glucocorticoid therapy followed by steroid-sparing agents such as azathioprine, methotrexate, and mycophenolate mofetil to control disease activity. Refractory myositis can be defined as inadequate response to at least two steroid-sparing agents for a minimum period of 3 months.^1^ In this setting, to minimize disease burden, avoid end-organ damage and steroid toxicity, and decrease mortality, second-line therapies are needed. These may include biological disease-modifying antirheumatic drugs (bDMARDs) and targeted synthetic DMARDs (TSD). This article reviews the current use of bDMARDs and TSD, including their indications, efficacy, and safety, in managing IIM.

## INTRAVENOUS IMMUNOGLOBULIN USE IN IIM

Intravenous immunoglobulin (IVIg), a purified liquid IgG concentrated from human plasma, has been approved by the US Food and Drug Administration (FDA) for treating adults with dermatomyositis (DM). In high-risk patients (e.g. dysphagia, severe weakness), IVIg may be used as a first-line treatment. Several mechanisms of action have been proposed for the beneficial effect of IVIg in IIM. These include: (1) inhibition of complement activation and capillary membrane attack complex (MAC) deposition;^2^,^3^ (2) downregulation of genes related to inflammation, e.g. IL-2, KAL-1, ICAM-1, C1q;^4^ (3) upregulation of chemokines CXCL9 and CXCL11-related genes;^4^ and, finally, (4) blocking the Fc-receptors on autoantibodies that prevent antibody-coated cell phagocytosis.^5^,^6^

In 1993 Dalakas et al. reported that IVIg improved muscle strength and reduced neuromuscular symptoms in a randomized controlled trial of 15 patients.^7^ Later on, in an open-label study from 2002 in which IVIg was used in 35 patients, 50% showed marked improvement in disease activity, with durable efficacy over three years.^8^ In Aggarwal et al.’s recent randomized controlled trial (ProDERM trial), refractory IIM patients or those receiving concomitant glucocorticoid and immunosuppressive medication saw significant improvement with IVIg, administered at a dose of 2.0 g per kg of body weight. At 16 weeks, 79% of the patients in the IVIg group (37 of 47) and 44% of those in the placebo group (21 of 48) showed at least slight improvement on a composite score of disease activity (*P*<0.001). Additionally, the median time to at least modest improvement with IVIg was 35 days, while the median time with placebo was 115 days.^9^ Intravenous Ig was also reported to be highly effective in the setting of myositis-related dysphagia, a life-threatening myositis manifestation. In a retrospective analysis, 12 of 18 patients treated with IVIg for refractory dysphagia had completely recovered by week 52.^10^ Furthermore, IVIg was reported to be particularly effective in treating cutaneous dermatomyositis; a retrospective study included 42 patients with refractory cutaneous dermatomyositis treated with IVIg and showed that 57% of patients with cutaneous DM responded after one IVIg cycle, and 80% showed improvement after two IVIg cycles, regardless of sex, smoking status, DM subtype, the reason for IVIg initiation, days from DM diagnosis to IVIg initiation, specific cutaneous manifestations, or serological finding. As a result, patients were able to taper off of steroids and discontinue steroid-sparing immunosuppressive agents.^11^ Finally, a recent meta-analysis by Goswami et al. included 29 studies (a total of 576 patients treated with IVIg). They reported a pooled muscle power improvement with at least a partial response rate of 77.07% with first-line use of IVIg according to the International Myositis Assessment and Clinical Studies (IMACS) measure. The overall mean time to response was 2.9 months, with a significant treatment response on cutaneous disease activity and dysphagia. The steroid and immunomodulating agents sparing effect was reported to be 40.9%.^12^

In terms of safety, in addition to the well-known adverse effects of IVIg, including nausea, headaches, and fever, venous thromboembolism (VTE) is of particular concern since both IVIg treatment and inflammatory myopathy may increase the risk for VTE.^13^–^16^ In the ProDERM trial, six patients treated with IVIg experienced eight thromboembolic events (an incidence of 1.54 occurrences per 100 patient-months). This observation led to a protocol change in IVIg infusion rate in order to minimize thromboembolic incidence.^9^

## RITUXIMAB USE IN IIM

Rituximab is a chimeric anti-CD20 monoclonal antibody used to treat various diseases such as rheumatoid arthritis and vasculitis. Juvenile DM (JDM), characterized by substantial increases in type I interferon (IFN) and immature transitional B cells, provides evidence for the involvement of B cells in the etiology of IIM.^17^ Furthermore, the serum and muscle fibers of IIM have higher levels of B cell activating factor, a member of the tumor necrosis factor (TNF) family.^18^ These findings support the hypothesis that B-cell depletion may reduce the severity of IIM and have a beneficial effect on its disease burden. A hallmark study regarding rituximab use in IIM, the Rituximab in Myositis (RIM) trial, was a randomized placebo-phase controlled clinical trial of adult patients with refractory IIM. A total of 200 patients were enrolled in the “rituximab early” or “rituximab late” arm of the study 8 weeks later. Even though there were no significant differences between the treatment groups in the time (20 weeks) of achieving the definition of improvement (DOI) based on IMACS, up to 83% of the study patients achieved the DOI at week 44 and were able to taper their glucocorticoid therapy with an excellent general response rate.^19^ A recent meta-analysis, which included 26 studies and almost 450 patients with IIM, showed that the overall efficacy rate of rituximab was 65%; a complete response rate of 35% was reported in seven trials (121 patients), with improvement in muscle strength, skin involvement burden, and pulmonary function tests/radiographic lung findings in the majority of patients.^20^

The anti-synthetase syndrome (ASS) is a subcategory of IIM; according to a study of Allenbach et al., only 20% of refractory ASS patients treated with rituximab achieved a primary endpoint of an increase in muscle strength, yet other studies demonstrated a pooled effectiveness of 62% of rituximab in ASS.^20^,^21^

Interestingly an observational study demonstrated that patients with IIM treated with rituximab had fewer and milder side effects compared to patients with rheumatoid arthritis and systemic lupus erythematosus.^22^ The rituximab dosage is crucial because of cost and the raised susceptibility to opportunistic infections and viral infections (e.g. COVID-19). Hence some investigators evaluated the efficacy of low and ultra-low rituximab doses for IIM. One study by Janardana et al. showed that a 0.5 g + 0.5 g rituximab regimen (2 weeks apart) had a similar effect to the 1 g + 1 g regimen.^23^ Mao et al. used an ultra-low dose of rituximab (100 mg) as an add-on therapy for patients with anti-MDA5-positive interstitial lung disease (ILD), resulting in persistent B-cell depletion that lasted 180 days, and may decrease mortality.^24^

## JANUS KINASE INHIBITOR USE IN IIM

The Janus kinase/signal transducers and activators of transcription (JAK/STAT) pathway has a significant role in signaling inflammatory cytokines and immunoregulation. Activation of JAK1/2 induces phosphorylation of STAT1, a key transcription factor that mediates IFN-I signaling. In myositis, upregulation of type I IFN-regulated genes in peripheral blood, muscle, skin, and endothelial cells and elevated serum IFN-a serum correlates with disease activity.^25^,^26^ It also seems that IFN-I has a crucial role in the pathogenesis of myopathy, and one study showed that ruxolitinib inhibits the pathogenic effects of IFN-I in both muscle and endothelial cells.^27^

One of the early indicators for the effectiveness of Janus kinase inhibitors (JAKI) in IIM came from a case report describing a 72-year-old woman with severe muscle and cutaneous DM, diagnosed with a JAK2-V617F mutation-positive myelofibrosis one year after her DM diagnosis. She was treated with ruxolitinib for myelofibrosis, resulting in rapid resolution of her muscle and skin symptoms. Glucocorticoids, mycophenolate mofetil, and IVIg were tapered and eventually discontinued.^28^ Kim et al. reported four patients with refractory JDM treated with baricitinib 4 mg daily, with significant improvement by week 4 in muscle and skin manifestations, with no serious adverse events reported.^29^ In addition, in a retrospective study that included 10 patients with either new-onset or refractory JDM, particularly anti-MDA5- or anti-NXP2-positive, treatment with ruxolitinib or baricitinib resulted in clinically inactive disease within 6 months in 50% of patients.^30^ Evidence for the efficacy of tofacitinib was reported initially in a case series of four patients with refractory DM who responded well to tofacitinib with improvement in their cutaneous, muscle, and joint symptoms.^31^ Furthermore, in an open-label prospective clinical trial 10 patients with skin-predominant DM and at least moderate skin disease activity by the Cutaneous Dermatomyositis Disease Area and Severity Index (CDASI) were treated with tofacitinib; there was moderate improvement in disease activity in 5 patients according to the IMACS group DOI and a mean reduction in the CDASI score of 18.5.^32^ Finally, a review of 14 studies including 53 patients with refractory DM showed substantial durable improvement in cutaneous skin signs and muscle strength after using JAKI with the ability to taper down the steroid dose in the majority of patients.^33^

Although the most reported adverse effect of JAKI was mild-to-moderate herpes zoster infection, VTE is of particular concern, since both IIM and JAKI are associated with increased risk for VTE.^29^–^32^

## ABATACEPT USE IN IIM

The predominance of T cells in the inflammatory infiltrates in DM and polymyositis muscle biopsies is evidence for the role of T cells in its pathogenesis. Furthermore, increased CTLA-4, CD28, CD86, and CD40 expression has been described.^34^

Abatacept, a fully human fusion protein of CTLA-4 and the Fc portion of human IgG1, is a physiological antagonist of the T cell co-stimulatory molecule CD28.^35^ Research conducted by Tjärnlund et al. showed that 42% of patients with myositis who were treated early with abatacept had lower disease activity according to the IMACS group DOI, with significant improvement in muscle performance as evidenced by improved manual muscle tests (MMT) 8 score, a validated tool based on assessment of the strength of eight muscle groups.^36^ Post hoc analysis showed an interesting finding of a positive correlation between the CD4/CD8 ratio in peripheral blood samples at baseline and improved muscle endurance after treatment; however, no significant changes in circulating T and B cell levels were observed.^37^

Abatacept appears to be a relatively safe biologic. Its most frequently reported adverse event is upper respiratory tract infections, followed by cardiovascular effects, all considered mild or moderate in severity.

## TOCILIZUMAB USE IN IIM

Interleukin-6 acts as a mediator of muscle inflammation. Dysregulated IL-6 production has been shown to contribute to the pathogenesis of DM in preclinical studies. Furthermore, tocilizumab, an IL-6 receptor antagonist, had favorable effects on myositis in mouse models.^38^–^40^ Serum IL-6 levels in adult and juvenile DM have been shown to parallel disease activity, and a small number of patients with refractory polymyositis have responded favorably to treatment with tocilizumab.^41^,^42^ Further evidence of tocilizumab tolerability was shown in a prospective phase IIb clinical trial where 36 patients with myositis were randomized 1:1 to receive tocilizumab or a placebo every 4 weeks for 24 weeks. Tocilizumab was well tolerated but not more effective than the placebo.^43^ Conversely, in a case-control study with 11 patients with refractory immune-mediated necrotizing myopathy, including 3 with anti-3-hydroxy-3-methyl glutaryl-CoA reductase and positive anti-signal recognition particles, 63% achieved clinically significant responses.^44^ Responders had higher baseline serum IL-6 and muscle IL-6 mRNA levels and higher percentages of CD56-positive muscle fibers than did non-responders. Another study showed the beneficial effect of tocilizumab as a third-line biologic in patients with refractory ASS.^45^,^46^ Yet another case-control study using anti-IL-6 suggested that tocilizumab may be used as a salvage therapy for rapidly progressive interstitial lung disease patients refractory to an intensive immunosuppressive regimen.^47^

## MYOSITIS AND ANTI-TUMOR NECROSIS FACTOR

As inhibiting TNF increases type I IFN production, the use of TNF blockers may be a potential trigger for developing or exacerbating inflammatory myopathy.^48^,^49^ Although some case reports show a beneficial effect of anti-TNF in myositis, especially in joint or skin predominant disease,^50^,^51^ data from retrospective studies reveal an increased risk for ASS and exacerbation of IIM-related ILD.^52^

## LENABASUM AND DERMATOMYOSITIS

Lenabasum, a non-immunosuppressive, non-psychoactive cannabinoid type 2 receptor reverse agonist, is an agent recently investigated in the settings of dermatomyositis.^53^ Activation of the cannabinoid type 2 receptor has been shown to reduce several vital pro-inflammatory cytokines implicated in DM.^53^ In a recent double-blind, randomized, placebo-controlled trial, 22 patients were randomized to receive lenabasum or placebo. Lenabasum treatment was associated with more remarkable improvement in the CDASI activity. No serious adverse events were related to lenabasum.^54^

## USE OF BIOLOGIC DMARDS IN MYOSITIS-ASSOCIATED ILD

In addition to the use of bDMARDs in refractory inflammatory myopathy discussed above, interest in the use of these agents in the settings of myositis syndromes with lung involvement is increasing. Although the prevalence, course, histopathology, and severity of myositis-associated ILD vary widely because of many factors, several subtypes, such as amyopathic dermatomyositis, MDA5, and ASS, warrant particular concern.

Intravenous Ig has been reported to be effective in several cases and case series in the settings of progressive ILD associated with myositis. It has also been shown to be effective in cases of rapidly deteriorating MDA5 syndrome.^55^–^58^

Data are accumulating from case series regarding the use of rituximab in patients with progressive ILD with polymyositis or DM, including ASS.^59^–^62^ Furthermore, the RECITAL trial showed that rituximab seems to be as effective as cyclophosphamide for myositis-associated ILD, with fewer adverse events.^63^ Other case reports of patients with MDA5 antibodies described improvement in ILD with rituximab after the failure of other immunosuppressive therapies.^64^,^65^

In addition, in a case series of patients with myositis-associated ILD, 26 patients treated with tofacitinib were compared to 35 patients treated with tacrolimus. The 6-month and 1-year mortality rates were significantly lower in the tofacitinib group.^66^ Furthermore, an open-label study comparing tofacitinib JAKI to a standard regimen of immunomodulating agents among patients with MDA5 antibodies demonstrated a significant (*P*=0.04) improvement in survival at 6 months in the tofacitinib group (18 patients with 100% survival) compared to 78% survival (25 of 32 patients). Improved diffusing capacity of carbon monoxide and high-resolution computed tomography findings were also seen with tofacitinib.^67^

In recent years, antifibrotic medications, a group of well-studied agents in treating idiopathic interstitial pulmonary fibrosis,^68^,^69^ e.g. nintedanib and pirfenidone, were used to treat autoimmune-associated ILD including myositis-associated ILD. In a retrospective, real-world analysis of IIM-ILD patients, nintedanib, a tyrosine kinase inhibitor, appeared to be protective against the development of rapidly progressive ILD and was associated with improved survival in myositis-associated ILD.^70^ Similar beneficial effects were shown in a prospective controlled cohort study conducted by Wang et al., in which pirfenidone showed an improvement in pulmonary function tests, imaging findings, and mortality in IIM-ILD patients.^71^

## CONCLUSION

Inflammatory myopathy syndromes are relatively rare disorders. Despite the lack of large cohort randomized controlled trials, in the past several years there has been more solid evidence regarding the use of advanced biologic therapy (e.g. IVIg and rituximab) based on the understanding of the pathogenic mechanisms of these syndromes. Use of JAKI also appears to be promising. While many patients may be managed in the ambulatory setting, acutely ill patients with active myositis or exacerbating lung disease must be managed in hospital and, in some cases, in the medical intensive care unit. To optimize high-quality care, these patients should be managed by a skilled multi-disciplinary team with rheumatologists, chest medicine specialists, and dermatologists.

Nevertheless, despite advances in treating IIM in the past years, much remains unknown and special efforts should be made to offer more precise therapy based on clinical presentation, organ involvement, and biochemical markers.

## Abbreviations

bDMARDs: biologic disease-modifying antirheumatic drugs
DM: dermatomyositis
DOI: definition of improvement
DMARDs: disease-modifying antirheumatic drugs
IFN: interferon
IIMs: idiopathic inflammatory myopathies
IMACS: International Myositis Assessment and Clinical Studies
IVIg: intravenous immunoglobulin
JDM: juvenile dermatomyositis
JAK: Janus kinase
JAKI: Janus kinase inhibitors
STAT: signal transducers and activators of transcription
TNF: tumor necrosis factor
TSD: targeted synthetic disease-modifying antirheumatic drugs
VTE: venous thromboembolism.
